# Mutational and Structural Analysis of KIR3DL1 Reveals a Lineage-Defining Allotypic Dimorphism That Impacts Both HLA and Peptide Sensitivity

**DOI:** 10.4049/jimmunol.1303142

**Published:** 2014-02-21

**Authors:** Geraldine M. O’Connor, Julian P. Vivian, Jacqueline M. Widjaja, John S. Bridgeman, Emma Gostick, Bernard A. P. Lafont, Stephen K. Anderson, David A. Price, Andrew G. Brooks, Jamie Rossjohn, Daniel W. McVicar

**Affiliations:** * Cancer and Inflammation Program, Center for Cancer Research, National Cancer Institute, Frederick, MD 21702;; † Department of Biochemistry and Molecular Biology, School of Biomedical Sciences, Monash University, Clayton, Victoria 3800, Australia;; ‡ Department of Microbiology and Immunology, University of Melbourne, Parkville, Victoria 3010, Australia;; § Institute of Infection and Immunity, Cardiff University School of Medicine, Heath Park, Cardiff CF14 4XN, United Kingdom;; ¶ Non-Human Primate Immunogenetics and Cellular Immunology Unit, Laboratory of Molecular Microbiology, National Institute of Allergy and Infectious Diseases, National Institutes of Health, Bethesda, MD 20892;; ‖ Basic Science Program, Leidos Biomedical Research Inc., Laboratory of Experimental Immunology, Frederick National Laboratory, Frederick, MD 21702; and; # Human Immunology Section, Vaccine Research Center, National Institute of Allergy and Infectious Diseases, National Institutes of Health, Bethesda, MD 20892

## Abstract

Killer Ig-like receptors (KIRs) control the activation of human NK cells via interactions with peptide-laden HLAs. KIR3DL1 is a highly polymorphic inhibitory receptor that recognizes a diverse array of HLA molecules expressing the Bw4 epitope, a group with multiple polymorphisms incorporating variants within the Bw4 motif. Genetic studies suggest that KIR3DL1 variation has functional significance in several disease states, including HIV infection. However, owing to differences across KIR3DL1 allotypes, HLA-Bw4, and associated peptides, the mechanistic link with biological outcome remains unclear. In this study, we elucidated the impact of KIR3DL1 polymorphism on peptide-laden HLA recognition. Mutational analysis revealed that KIR residues involved in water-mediated contacts with the HLA-presented peptide influence peptide binding specificity. In particular, residue 282 (glutamate) in the D2 domain underpins the lack of tolerance of negatively charged C-terminal peptide residues. Allotypic KIR3DL1 variants, defined by neighboring residue 283, displayed differential sensitivities to HLA-bound peptide, including the variable HLA-B*57:01–restricted HIV-1 Gag-derived epitope TW10. Residue 283, which has undergone positive selection during the evolution of human KIRs, also played a central role in Bw4 subtype recognition by KIR3DL1. Collectively, our findings uncover a common molecular regulator that controls HLA and peptide discrimination without participating directly in peptide-laden HLA interactions. Furthermore, they provide insight into the mechanics of interaction and generate simple, easily assessed criteria for the definition of KIR3DL1 functional groupings that will be relevant in many clinical applications, including bone marrow transplantation.

## Introduction

Killer Ig-like receptors (KIRs) are a rapidly evolving family of transmembrane glycoproteins expressed on subsets of T cells and NK cells in humans. Fourteen constituent members are described, including both inhibitory and activating receptors (1). At present, the best characterized function of KIRs is the transmission of an inhibitory signal upon engagement of HLA molecules.

The three Ig domain (D0, D1, D2) inhibitory receptor 3DL1 recognizes HLA-A and HLA-B molecules that express the Bw4 public epitope (2) within the α1 domain of the HLA H chain. More than 70 allotypes of 3DL1 have been described to date, in addition to the presence of an activating receptor variant termed 3DS1 (3). Analysis of genetic sequence variation has led to the grouping of *3DL1* alleles into three lineages, *3DS1*, *015-like, and *005-like alleles, which appear to have been maintained through evolution via balancing selection (4). 3DL1 polymorphism can impact allotype expression levels, HLA recognition, and peptide sensitivity (5–9). Based on Ab staining of peripheral blood NK cells, 3DL1 variants were initially grouped into “high”- (e.g., *015, *001) and “low”- (e.g., *005) expressing allotypes (6, 10). It has now become clear that there is a spectrum of cell surface expression levels primarily dictated by the degree of intracellular KIR retention (11). Specific interactions with HLA molecules are also allotype-dependent (5, 8). Additionally, tetramer binding studies have uncovered a role for allotypic diversity in peptide selectivity (9). Although some polymorphic residues in 3DL1 have been shown to affect the strength of the inhibitory signal after HLA engagement (e.g., the D2 residue 238 and the transmembrane residue 320 in 3DL1*007), the molecular mechanisms responsible for these functional differences remain unclear (5).

Originally defined serologically, the Bw4 epitope is formed by amino acid residues at positions 77–83 on the α1 helix of the HLA molecule. Within the Bw4 epitope itself, there are three polymorphic positions (77, 80, and 81) giving rise to at least four distinct protein sequences. Notably, the dimorphic residue at position 80 incorporates either isoleucine (Bw4 80I) or threonine (Bw4 80T). This position was identified as a point of contact in the recently reported 3DL1*001-HLA-B*57:01 structure, and mutation of Ile to Thr at position 80 of HLA-B*57:01 reduced the KIR–HLA interaction affinity (12). Earlier reports also provide evidence that 3DL1 prefers HLA Bw4 molecules that express isoleucine rather than threonine at position 80 (13, 14).

The functional consequences of KIR–HLA interactions have been examined in genetic association studies across many disease cohorts (15). In HIV infection, Martin et al. (16) reported that distinct *KIR/HLA* combinations are beneficial. Specifically, genes encoding high *3DL1* allotypes provided greater protection from progression to AIDS when found in combination with *Bw4 80I* alleles (including HLA-B*57:01), whereas low 3DL1 variants enhanced protection associated with the Bw4 80T allele HLA-B*27:05. One possible interpretation of this dichotomy is that these KIR/HLA combinations interact potently to deliver optimal inhibitory signals to immune cells. Loss of this signal owing to HIV-induced HLA class I downregulation, infection-related changes in peptide presentation patterns, or other mechanisms would consequently result in greater levels of effector cell activation. Associations based on similar KIR groupings (high versus low) have also been found in studies of susceptibility to HIV acquisition (17) and leukemia relapse after allogeneic hematopoietic stem cell transplantation (Katharine Hsu, Memorial Sloan–Kettering Cancer Center, personal communication). Although these results highlight the role of NK cells and 3DL1 in disease outcomes, the underlying functional mechanisms have not been explored.

We recently described the crystal structure of 3DL1*001 in complex with HLA-B*57:01 presenting a self-peptide (12). The D0 domain, a previously poorly understood feature of KIR–HLA interactions, contacted a conserved region of HLA-B*57:01 extending toward the invariant β_2_-microglobulin molecule, thereby acting as an “innate” HLA binding domain. In contrast, the D1–D2 binding face contacted the Bw4 epitope of HLA-B*57:01 and position 8 (P8) of the bound peptide. These structural data support previous studies in which the interaction of 3DL1 with HLA-B*27:05 was found to be dependent on the peptide residues at P7 and P8 (18–20). The functional role of peptide selectivity was further highlighted by Liberatore et al. (21) who found that transduction with a neo-expression construct rendered cells susceptible to lysis by autologous NK cells. This effect was due to a loss of 3DL1 recognition resulting from preferential loading of a nonpermissive peptide into HLA-B*27. Such perturbations of the HLA-bound peptide repertoire may arise naturally due to changes in intracellular protein content (e.g., during infection, transformation, or cellular stress) or iatrogenically due to drug-mediated effects on peptide-binding profiles (22). Accordingly, 3DL1 recognition of a limited HLA-bound peptide repertoire provides a mechanism for immune cell activation as a consequence of changes in cellular processes associated with infection or transformation.

Despite clear evidence from genetic studies that Bw4 80T/80I discrimination is fundamental to certain disease outcomes, we do not yet fully understand the mechanisms involved at the molecular level or the role of 3DL1 polymorphism in this process. In this study, we performed extensive binding and mutational analyses in conjunction with structural investigations to decipher the functional consequences of allelic variation at the *3DL1* locus.

## Materials and Methods

### Cell lines

HEK293T cells were maintained in DMEM supplemented with 10% FCS, 2 mM l-glutamine, 50 U/ml penicillin, and 50 μg/ml streptomycin. Jurkat cells stably expressing chimeric 3DL1-CD3ζ reporter constructs (8) were maintained in RPMI 1640 medium supplemented with 10% FCS, 2 mM l-glutamine, 50 U/ml penicillin, 50 μg/ml streptomycin, and 0.5 mg/ml geneticin. Ba/F3 cells were maintained in RPMI 1640 medium supplemented with 10% FCS, 2 mM l-glutamine, 50 U/ml penicillin, 50 μg/ml streptomycin, and 10 ng/ml murine IL-3. RMA-S cell lines were maintained in DMEM supplemented with 10% FCS, 2 mM l-glutamine, 50 U/ml penicillin, 50 μg/ml streptomycin, and 0.5 mg/ml geneticin.

### Crystallization and data collection

The HLA class I H chain and β_2_-microglobulin were refolded from inclusion body preparations expressed in *Escherichia coli* and purified as detailed previously (23). KIR3DL1*001 was expressed in HEK293S cells and purified from the secreted fraction by nickel-affinity and size-exclusion chromatography. Crystals were obtained at 294 K by the hanging-drop vapor-diffusion method. The ternary complex was crystallized in conditions previously established (12) from a reservoir solution comprising 16% PEG 3350, 0.1 M trisodium citrate (pH 6), and 4% Tacsimate (pH 5). Prior to data collection the crystals were flash-cooled in a stream of liquid nitrogen at 100 K in a cryoprotectant composed of reservoir solution supplemented with 35% PEG 3350. X-ray diffraction data were recorded on a Quantum-315 CCD detector at the MX2 beamline of the Australian Synchrotron. Data were integrated and scaled using MOSFLM and SCALA from the CCP4 program suite (24). Details of the data processing statistics are provided in Table I.

### Structure determination and refinement

Structures were determined by molecular replacement as implemented in PHASER (25). The search model used for the ternary complex was the previously determined structure of the KIR3DL1-B57:01-LF9 ternary complex (Brookhaven Protein Data Bank accession code 3VH8). Refinement of the models was carried out in PHENIX (26) with iterative rounds of manual building in COOT (27). Solvent molecules were added with COOT and the structure validated with MOLPROBITY (28). The final refinement values are summarized in Table I.

### Mutagenesis and transfection studies

3DL1 constructs with a 5′ FLAG tag sequence (GACTACAAAGACGATGACGACAAG) were cloned into a pEF6 vector. Specific nucleotide residues were mutated using a QuikChange II site-directed mutagenesis kit (Stratagene) with PAGE-purified primers and verified by direct sequencing. These constructs were introduced into HEK293T cells using FuGENE 6 transfection reagent (Roche) according to the manufacturer’s instructions. Expression, localization, and correct folding of KIR proteins were monitored by flow cytometry after staining with DX9, Z27, and anti-FLAG mAbs.

### Tetramer staining

Fluorochrome-conjugated peptide-HLA class I tetramers were produced as described previously (29). The specificities used in this study are detailed in Table II. Cells were stained 48 h after transfection with optimal titers of tetramer (0.2 μg with respect to the monomeric component in minimal residual volume) or anti-FLAG (clone M2; Sigma-Aldrich) mAb for 30 min at 4°C. Ba/F3 cells stably expressing LILRB1 were used as a positive control for tetramer binding. The LILRB1 receptor binds with equivalent affinity to a broad range of HLA molecules, and this binding is dependent on the correct folding of the HLA molecule and its association with β_2_-microglobulin (30), but is independent of the presented peptide (31). Primary human PBMCs were stained similarly with 0.5 μg tetramer, anti-CD3 (clone HIT3a; BD Biosciences), and anti-CD56 (clone HCD56; BioLegend) mAbs. After RBC lysis in hypotonic buffer, cells were washed and analyzed using an LSRFortessa flow cytometer (BD Biosciences). For blocking experiments, cells were preincubated with the indicated mAb (10 μg/ml) for 15 min at 4°C.

### Peptide loading and activation experiments

RMA-S cells transfected with HLA-B*5701 were incubated with individual peptides at a concentration of 300 μM for 24 h at 26°C. Stabilization of HLA class I was measured by staining with W6/32 and anti-HLA-B/C (B1.23.2) mAbs (eBioscience). Jurkat cells stably expressing chimeric 3DL1-CD3ζ reporter constructs (8) were transiently transfected with an NFAT-luciferase construct by electroporation, then stimulated with peptide-loaded RMA-S cells for 18 h at 26°C. Luciferase activity was measured after cell lysis using a Dual-Glo luciferase assay (Promega).

3DL1*001-CD3ζ and 3DL1*005-CD3ζ chimeric constructs were mutated at position 283 as described above. Jurkat cells were transiently transfected with these KIR constructs together with an NFAT-luciferase reporter construct, then incubated for 18 h at 37°C with parental 721.221 cells or specific HLA class I transfectants thereof (B*27:05, B*44:02, and B*57:01). Luciferase activity was measured after cell lysis as described above.

## Results

### Role of contact sites in the sensitivity of 3DL1*001 to C-terminal peptide variation

**FIGURE 1.**
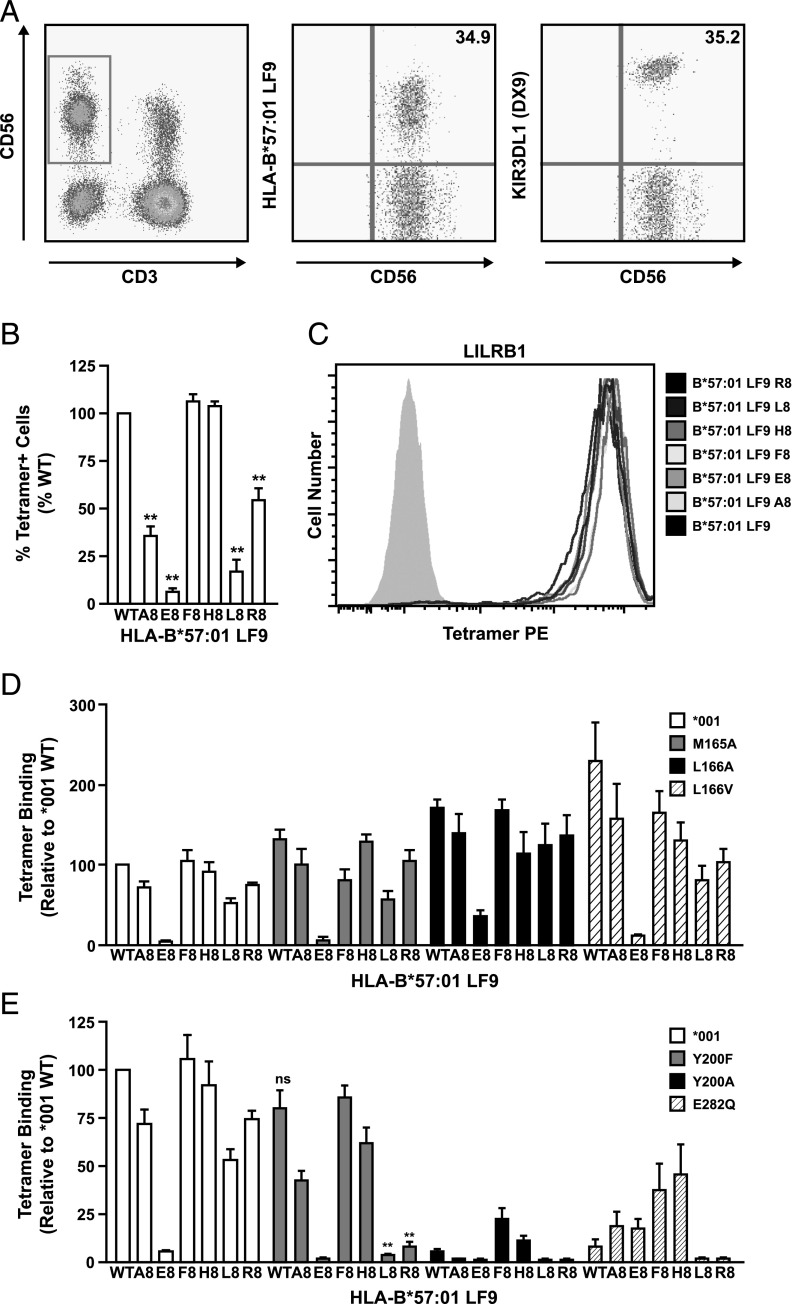
Amino acid residues at peptide position P8 influence 3DL1*001 binding to HLA-B*57:01. (**A**) Peripheral blood from a 3DL1*001^+^ donor was stained with HLA-B*57:01-LF9 tetramer (0.5 μg) and mAbs as indicated. (**B**) Peripheral blood NK cells were stained as in (A) with individual HLA-B*57:01-LF9 tetramers incorporating bound peptide substitutions at position P8. Data are shown as the percentage of positive cells relative to wild-type (WT) tetramer staining. (**C**) Ba/F3 cells expressing LILRB1 were stained with the same panel of tetramers. (**D** and **E**) HEK293 cells were transfected with FLAG-tagged 3DL1*001, or single amino acid variants thereof, and stained with individual HLA-B*57:01-LF9 tetramers (0.2 μg each) as in (B). Data are averaged from *n* = 2 (D) or *n* = 3 (B, E) independent experiments. **p < 0.01. Statistical significance relative to 3DL1*001 binding was assessed by ANOVA with a Tukey multiple comparison posttest. Error bars represent SE of the mean.

The recently published crystal structure of 3DL1*001 in complex with HLA-B*57:01-LSSPVTKSF (LF9) highlighted an important role for the P8 C-terminal residue of the bound peptide in the KIR–HLA interaction. In line with the structural evidence, surface plasmon resonance (SPR) experiments showed that variation at P8 affected the affinity of the interaction between HLA-B*57:01 and 3DL1*001 (12). To extend this analysis, we examined the peptide sensitivity of 3DL1*001 expressed on human NK cells. Peripheral blood NK cells from 3DL1*001^+^ donors were identified in flow cytometry experiments by gating on CD3^−^CD56^+^ lymphocytes. Tetrameric HLA-B*57:01-LF9 complexes (tetramers) clearly stained a subset of these NK cells, mirroring the percentage of 3DL1*001^+^ cells (Fig. 1A). Moreover, tetramer staining could be blocked by incubation with the 3DL1-specific DX9 mAb (data not shown). We then examined the interaction of 3DL1*001 with a series of HLA-B*57:01 tetramers in which the P8 serine residue of LF9 was replaced in turn by alanine (A8), glutamic acid (E8), phenylalanine (F8), histidine (H8), leucine (L8), and arginine (R8) (Fig. 1B). The integrity of this tetramer panel was confirmed by staining LILRB1-expressing Ba/F3 cell transfectants (Fig. 1C). In contrast to the staining of LILRB1, 3DL1*001 binding was profoundly influenced by variation at the P8 residue. Similar to the findings using SPR (12), the larger, bulky residues, phenylalanine and histidine, were well tolerated. In contrast, the charged residues, arginine and glutamic acid, significantly affected KIR3DL1*001 binding; the glutamic acid substitution almost abrogated tetramer staining. More surprisingly, the relatively compact, nonpolar amino acids, alanine and leucine, also impaired KIR3DL1*001 binding. Although the pattern and hierarchy of interactions seen with the P8 tetramer panel is the same as that measured by SPR (12), there are some differences in the sensitivity; for example, whereas LF9-F8 and LF9-H8 show some reduction in affinity by SPR, they bind as well as the wild-type peptide when measured by flow cytometry. Based on SPR, the affinity for LF9-R8 drops below that of LF9-F8 and LF9-H8, and we now detect a dramatic loss in tetramer binding with this variant. This suggest that, based on the levels of KIR expression on NK cells, binding as detected by flow cytometry is relatively insensitive to small changes in *K*_d_, but tetramer binding decreases notably once the affinity drops below a certain threshold.

**Table I.** Data collection and refinement statistics for 3DL1*001-HLA-B*57:01.I80T-LF9 ternary complex

	KIR3DL1*001-HLA-B*57:01.I80T-LF9
Data collection statistics	
Temperature (K)	100
X-ray source	MX2 Australian synchrotron
Space group	*P*1
Cell dimensions (Å)	*a* = 52.1, *b* = 61.5,
	*c* = 66.4, α = 94.7°,
	β = 99.6°, γ = 109.1°
Resolution (Å)	40–2.00 (2.11–2.00)
Total no. observations	19,5349 (28,268)
No. unique observations	49,831 (7,177)
Multiplicity	3.9 (3.9)
Completeness (%)	98.2 (97.2)
1/σ_I_	8.4 (2.8)
* R*_merge_*^a^*	0.12 (0.66)
Refinement statistics	
Nonhydrogen atoms	
Protein	5,865
Water	493
* R*_factor_*^b^*	0.198
* R*_free_*^b^*	0.238
Root mean square deviation from ideality	
Bond lengths (Å)	0.008
Bond angles (°)	1.29
Ramachandran plot	
Favored (%)	97.1
Allowed (%)	2.8
B-factors (Å^2^)	
Average main chain	37.9
Average side chain	41.8
Average water	41.4

^*a*^ *R*_merge_ = ∑_hkl_ ∑_j_ |*I*_hkl,j_ − < *I*_hkl_ >|/∑_hkl_ ∑_j_ *I*_hkl,j_.

^*b*^ *R_factor_* = Σ_hkl_ ||*F*_o_| − |*F*_c_||/Σ_hkl_ |*F*_o_| for all data excluding the 5% that comprised the *R_free_* used for cross-validation.

**Table II.** List of tetramers used in this study

Name	HLA	Peptide	Peptide Source
LF9	B*57:01	LSSPVTKSF	Human
LF9 A8	B*57:01	LSSPVTKAF	Modified from human
LF9 E8	B*57:01	LSSPVTKEF	Modified from human
LF9 F8	B*57:01	LSSPVTKFF	Modified from human
LF9 H8	B*57:01	LSSPVTKHF	Modified from human
LF9 L8	B*57:01	LSSPVTKLF	Modified from human
LF9 R8	B*57:01	LSSPVTKRF	Modified from human
TW10	B*57:01	TSTLQEQIGW	HIV
TW10 G9D	B*57:01	TSTLQEQIDW	HIV
KK10	B*27:05	KRWIILGLNK	HIV
KF9	B*27:05	KRWGYSLNF	Hepatitis B virus
KF9 N8H	B*27:05	KRWGYSLHF	Hepatitis B virus
AF9	B*27:05	ARMILMTHF	Hepatitis C virus
RI9	B*27:05	RRFIIFLFI	Hepatitis C virus
AY10	B*44:02	AENLWVTVYY	HIV
RW8	A*24:02	RYPLTFGW	HIV
RW8 A8	A*24:02	RYPLTFAW	HIV
YL9	A*24:02	YLILEYAPL	Human
VL9	A*24:02	VYAETKHFL	Human
YI9	B*51:01	YPPPIQGVI	HIV

Structural data strongly implicated the direct peptide contact residue L166 and the water-mediated peptide contact residues Y200 and E282 as determinants of 3DL1 peptide selectivity. To test these structural predictions, we generated mutant 3DL1 proteins with single residue substitutions at these sites and examined their ability to bind HLA-B*57:01 tetramers refolded around LF9 or P8 variants thereof. Wild-type 3DL1*001 transfectants displayed tetramer binding preferences similar to those observed with primary NK cells (Fig. 1D), although with reduced sensitivity to disruption in the presence of unfavorable peptides, perhaps due to higher expression levels. The L166 residue forms the only direct contact with peptide in the HLA-B*57:01-LF9 complex, and variants are found at this site across members of the KIR family, including arginine in 3DS1 and valine in 3DL2. However, we found that the introduction of alanine or valine at position 166, or indeed alanine at position 165, only minimally affected 3DL1 peptide preferences (Fig. 1D). Although subtle and reproducible differences were observed, such as reduced binding to the LF9-F8 complex in the presence of the M165A mutation and increased tolerance of the LF9-L8 complex with L166A, these effects were minor.

Next, we addressed the role of Y200 and E282, which make water-mediated contacts with peptide in the HLA-B*57:01-LF9 complex. The Y200 residue is central to the 3DL1–peptide-laden HLA interaction and, as expected, the Y200A mutation substantially reduced binding across the series of HLA-B*57:01 tetramers. However, the LF9-F8 and LF9-H8 peptide variants partially rescued binding to this mutant (Fig. 1E). Although the Y200F substitution is more conservative, differing only by the absence of a hydroxyl group, this mutation resulted in a striking loss of tolerance for LF9-L8 and LF9-R8, whereas binding to other members of the tetramer panel was well maintained. The E282A mutation abolished interactions with HLA-B*57:01 across the entire panel of peptide variants (data not shown). In contrast, the E282Q mutation, which replaces the negatively charged glutamic acid with the similarly sized neutral glutamine, dramatically reduced HLA-B*57:01-LF9 tetramer binding. However, this was partially reversed in the presence of LF9-F8 and LF9-H8 (Fig. 1E). As predicted by the 3DL1*001-HLA-B*57:01-LF9 structure (12), removal of the charge at position 282 in 3DL1 resulted in a greater tolerance of LF9-E8. Similar to the Y200 substitutions, however, tolerance of LF9-L8 and LF9-R8 was completely lost with E282Q. Thus, we observed only minor effects due to substitutions at position 166, the point of direct peptide contact, but more dramatic shifts in relative peptide preference when residues in the D1–D2 loop (200) or D2 domain (282) were targeted.

### 3DL1 residues and variants distant from the peptide contact sites influence peptide preference

The ternary structure of the 3DL1*001-HLA-B*57:01-LF9 complex suggested that peptide preference should not be regulated by the D0 domain because its critical sites of interaction are distant from the peptide. To confirm this, we examined the binding of HLA-B*57:01-LF9 tetramers to mutant 3DL1 receptors bearing D0 substitutions at HLA contact residues. Although certain mutations of D0 residues involved in the HLA interaction (including positions 9, 11, and 29) did result in decreased HLA binding, we found that the pattern and hierarchy of reactivity against our peptide panel mirrored that of the wild-type KIR (data not shown). 3DL1*015 differs from *001 only in the D0 domain, with three amino acid differences (M2V, I47V, I54L). Despite the sequence divergence in D0, *015 revealed a strikingly similar binding profile to that of *001, both in terms of peptide selectivity and the role of D1/D2 amino acids (including L166, Y200, and E282) in this pattern of reactivity (data not shown). Collectively, these data suggest that D0 contacts and polymorphisms within the D0 domain play little or no role in peptide permissiveness.

**FIGURE 2.**
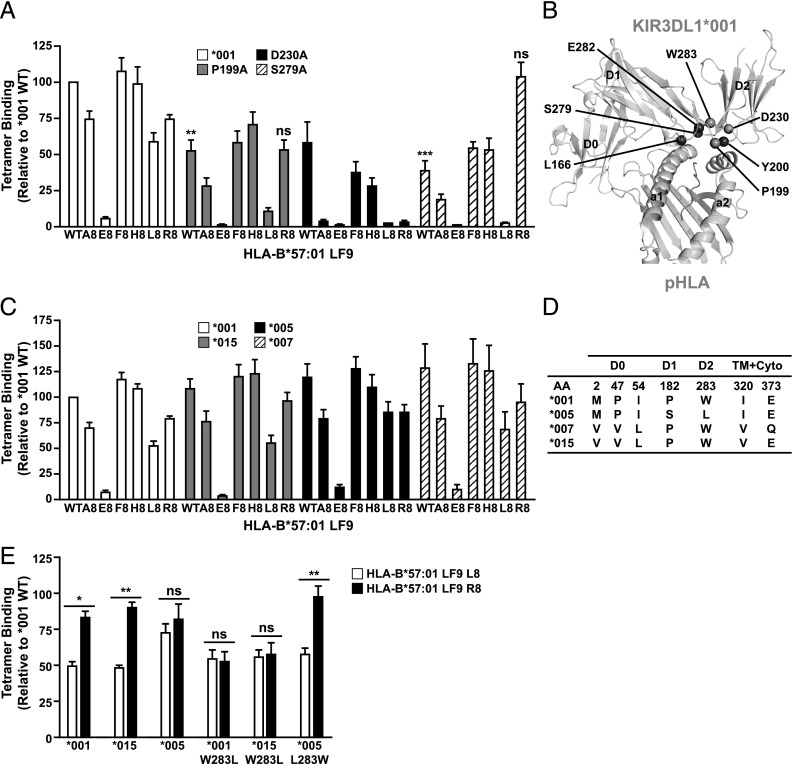
Allotypic variation influences peptide preference. (**A**, **C**, and **E**) HEK293 cells were transfected with FLAG-tagged 3DL1*001, other 3DL1 allotypes, or mutants thereof and stained with individual HLA-B*57:01-LF9 tetramers (0.2 μg each) as indicated. Data are averaged from at least *n* = 3 independent experiments. *p < 0.05, **p < 0.01. Statistical significance relative to 3DL1*001 binding was assessed by ANOVA with a Tukey multiple comparison posttest. Error bars represent SEM. (**B**) The structure of 3DL1*001-HLA-B*57:01-LF9 with residues of interest highlighted. (**D**) Alignment of the 3DL1 allotypes used in this study. Variable residues are numbered and grouped by protein domain in the mature full-length protein.

In addition to KIR residues involved in peptide contacts, our systematic analysis revealed that substitutions at other contact sites, including 199, 230, and 279, also modified 3DL1 peptide reactivity (Fig. 2A, 2B). The P199A mutation in the D1–D2 loop resulted in a minor decrease in overall HLA-B*57:01 recognition and also specifically reduced binding to the LF9-L8 tetramer (Fig. 2A). The D230A mutation resulted in a profound loss of reactivity for all but the three strongest interactions (LF9, LF9-F8, and LF9-H8). The S279A mutation, in contrast, resulted in a unique pattern of reactivity, with a very strong preference for the LF9-R8 tetramer. These changes in peptide specificity could be due either to direct effects on peptide interactions via alteration of the binding interface or indirect effects transmitted to other KIR residues.

Very few of the sites of 3DL1 allotypic variation map to the residues involved in the HLA-B*57:01 interaction, and all of the inhibitory 3DL1 variants (barring the unique *054) are identical at the residues involved in direct or water-mediated contacts with peptide (L166, Y200, and E282) (12). To explore how 3DL1 allotypic variation influences the repertoire of permissive peptides, we examined the ability of a range of 3DL1 allotypes to interact with our tetramer panel. Across a large panel of variants, we saw little effect on peptide preference (four common allotypes are shown in Fig. 2C, a full list of allotypes tested is shown in Supplemental Table I), with the exception of *005. This allotype showed a subtle shift in reactivity, with a slight reduction in LF9-R8 and an increase in LF9-L8 reactivity, such that both of these variants were equally well tolerated. 3DL1*005 differs from the other tested allotypes at position 283, which is located at the D1–D2 interface (Fig. 2B), carrying a leucine rather than a tryptophan (Fig. 2D). Because this residue is positioned directly beside the central E282, we examined the potential of L283 to influence peptide interactions. In two 3DL1 allotypes, *001 and *015, the single W283L mutation resulted in the *005-like phenotype with equivalent recognition of LF9-L8 and LF9-R8 (Fig. 2E). The reciprocal substitution, *005 L283W, resulted in a clear preference for LF9-R8 over LF9-L8, as observed with the *001 and *015 allotypes. In the case of L8, it is clear that in the context of *005, L8 binding is greater than that seen with *001 and *015, and that this difference is lost with the L283 mutation. In contrast, introduction of 283L into either *001 or *015 does not significantly increase L8 binding. This result suggests that additional changes in *005 (such as P182S) contribute to selective binding to peptide variants. Collectively, these data show that position 283 can impact the peptide sensitivity of 3DL1.

### Allotypic variation at position 283 in the D2 domain influences HIV-associated peptide variant recognition

**FIGURE 3.**
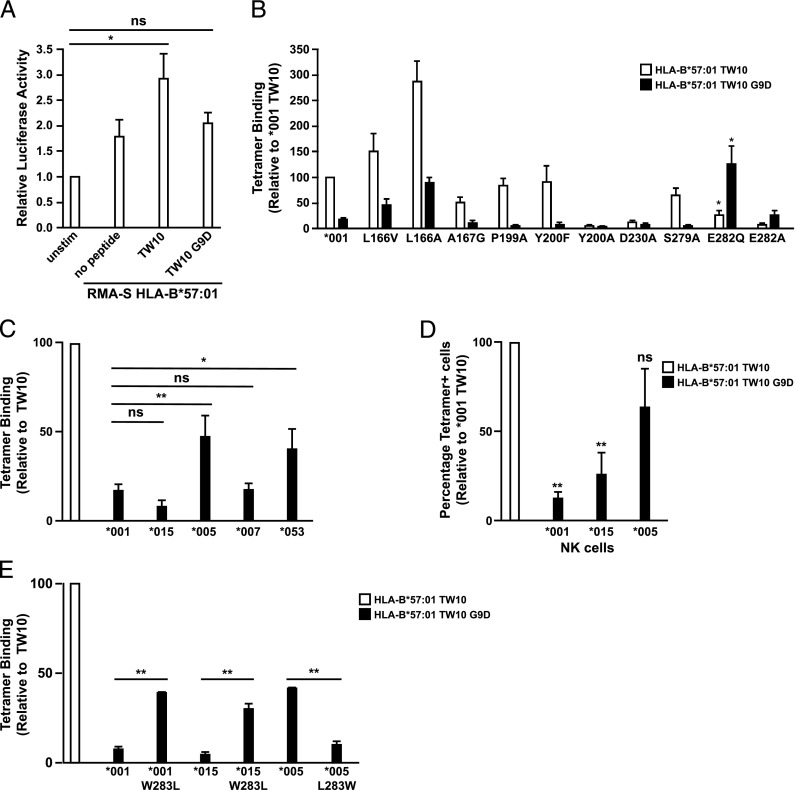
Position 283 in the D2 domain of 3DL1 allotypes dictates HIV-derived epitope variant recognition. (**A**) 3DL1*001-CD3ζ reporter Jurkat cells were stimulated with RMA-S HLA-B*57:01–expressing cells loaded with TW10 or TW10-D9 peptides for 18 h at 26°C. The cells were lysed and luciferase activity was measured. (**B** and **C**) HEK293 cells were transfected with FLAG-tagged 3DL1*001, other 3DL1 allotypes, or mutants thereof and stained with individual tetramers (0.2 μg each) as indicated. (**D**) Peripheral blood from 3DL1-genotyped donors was stained with individual tetramers (0.5 μg each) as indicated. Data are shown as percentage positive cells relative to those staining with the HLA-B*57:01-TW10 tetramer. (**E**) HEK293 cells were transfected with FLAG-tagged 3DL1 allotypes and mutants thereof with reverted amino acid substitutions at position 283, then stained with individual tetramers (0.2 μg each) as indicated. Data are averaged from at least *n* = 3 independent experiments. *p < 0.05, **p < 0.01. Statistical significance relative to KIR3DL1*001 binding was assessed by ANOVA with a Tukey multiple comparison posttest. Error bars represent SEM.

Based on our data with the LF9 peptide variants, we next examined whether these KIR contacts and polymorphisms played a role in the recognition of other peptides, specifically those presented during infection. Accordingly, we examined recognition of the HLA-B*57/B*58–restricted HIV-1 p24 Gag epitope TW10. The HLA-driven evolution of HIV-derived peptide sequences, including TW10, has been well documented (32). Described variants of TW10 include G9D, a residue predicted to alter 3DL1 interactions. In agreement with an earlier study (33), we found that this substitution did indeed result in a loss of 3DL1*001 binding (data not shown). To establish the functional consequences of altered peptide recognition in this system, we examined the effect of G9D substitution on 3DL1 triggering. A chimeric fusion protein consisting of 3DL1*001 fused to CD3ζ was expressed in Jurkat T cells and activation was measured with a luciferase reporter construct. Transfectants were then cultured alone or with the TAP-deficient RMA-S cell line expressing HLA-B*57:01 pulsed with either the TW10 or TW10-D9 peptide. Functional 3DL1*001 engagement was detected with TW10-pulsed cells, but not with TW10-D9–pulsed cells (Fig. 3A), despite equivalent stabilization of HLA-B*57:01 on the cell surface (data not shown). Thus, peptide discrimination toward the C terminus influences KIR engagement.

To uncover the mechanism responsible for the lack of HLA-B*57:01-TW10-D9 recognition, we conducted tetramer binding experiments with a series of 3DL1 mutants. We found that the loss of TW10-D9 tetramer binding was likely due to charge repulsion between the aspartic acid at P9 and E282 in the D2 domain of the KIR. Consistent with this, the E282Q substitution restored binding to levels observed with 3DL1*001 and the TW10 peptide (Fig. 3B). Indeed, in the presence of the E282Q mutation, the G9D peptide was vastly preferred over wild-type. These data indicate that the negative charge at position 282 plays a dominant role in discrimination of this variant epitope.

Although these findings shed light on the molecular rules that govern peptide selectivity, we wanted to address the role of naturally occurring variation given that position 282 is invariant across all 3DL1 allotypes. In light of the distinct peptide reactivity pattern observed with *005 in the context of LF9 variants (Fig. 2), we examined the ability of 3DL1 allotypes to bind TW10 and TW10-D9. Increased binding of the HLA-B*57:01-TW10-D9 tetramer by *005 relative to the *001 and *015 allotypes was observed on transfected cell lines (Fig. 3C) and primary human NK cells (Fig. 3D). Similar to our observations with the LF9 peptide panel, this pattern was not seen with *007, which is classified as a low allotype together with *005. However, *053, a poorly expressed allotype that contains a leucine at position 283 similar to *005, also exhibited substantial recognition of the G9D variant (Fig. 3C). These findings suggested that the observed pattern of peptide reactivity was not related to the low classification of these 3DL1 allotypes. Because we had demonstrated earlier that residue 283 was involved in LF9-L8/R8 discrimination, we tested TW10-D9 tetramer binding by *001, *015, and *005 allotypes in which position 283 was substituted with either tryptophan or leucine. The W283L mutation in both *001 and *015 resulted in a significant increase in TW10-D9 tetramer binding. Conversely, the L283W mutation in *005 significantly reduced TW10-D9 tetramer binding (Fig. 3E). Therefore, the presence of leucine at position 283 is specifically responsible for the recognition of HLA-B*57:01-TW10-D9. The importance of this residue potentially relates to modulatory effects on the nearby E282, which is responsible for the diminished reactivity with TW10-D9 due to charge repulsion.

### Position 283 controls 3DL1 allotypic discrimination between Bw4 80I and 80T

**FIGURE 4.**
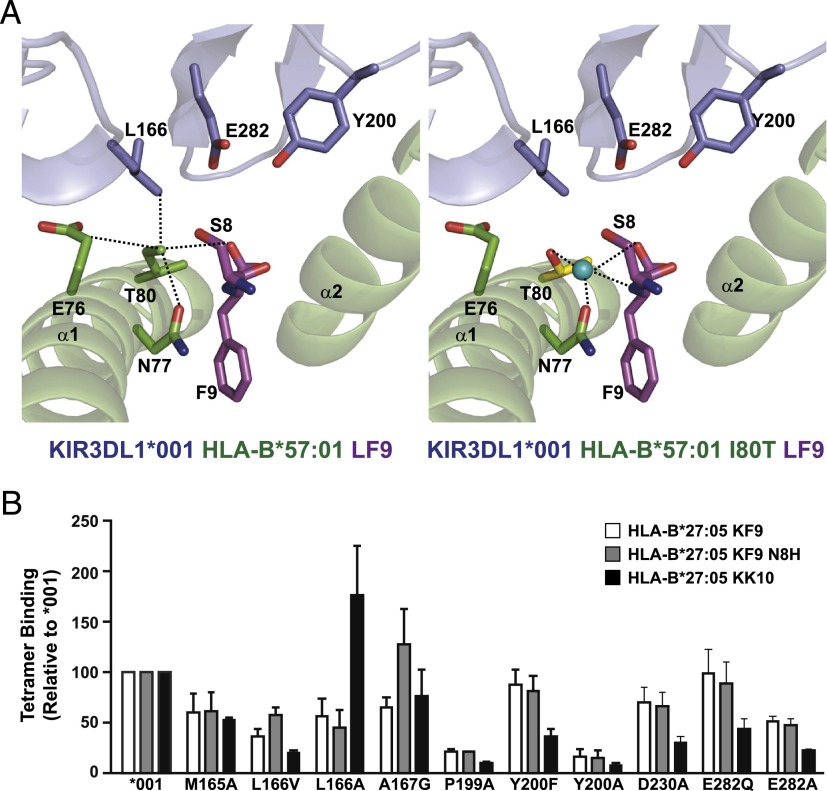
Interaction of 3DL1*001 with HLA-Bw4 80T allotypes strongly resembles the 3DL1/HLA-Bw4 80I interaction and is sensitive to peptide. (**A**) The crystal structure of HLA-B*57:01.I80T-LF9 in complex with 3DL1*001 (*right panel*) was solved and compared with the 3DL1*001-HLA-B*57:01-LF9 structure (*left panel*). The HLA and KIR are colored green and blue, respectively. Dashed lines represent contacts. (**B**) HEK293 cells were transfected with FLAG-tagged 3DL1*001 or mutants thereof, then stained with individual HLA-B*27:05 tetramers (0.2 μg each) as indicated.

A major division in HLA-Bw4 allotypes is based on the amino acid present at the dimorphic position 80 (isoleucine or threonine), and we have shown that the I80T mutation in the HLA-B*57:01-LF9 complex results in decreased binding to KIR3DL1*001 (12). This dichotomy within Bw4 molecules is of much interest owing to the resulting functional and clinical consequences reported by genetic studies (13, 16). To elucidate any unique features associated with the 3DL1–Bw4 80T interaction, we determined the structure of 3DL1*001 in complex with HLA-B*57:01 containing the I80T mutation to 2.0 Å resolution (Fig. 4A, Table I). The structures of the I80T mutant and the native complexes were very similar with a root mean square deviation over all Cα positions of 0.3 Å. Accordingly, the contacts between 3DL1 and the HLA were conserved with the exception of position 80. In the native ternary complex, the isoleucine at position 80 formed a network of van der Waals contacts via the C^δ1^ methyl group of the side chain (Fig. 4A). This network involved contacts to the carboxyl terminus of the peptide, the side chains of E76 and N77 (HLA), and L166 (KIR). These direct contacts were lost with the mutation to threonine at position 80. However, the network of contacts to the C^δ1^ group of I80 was partially restored in the T80 structure by an ordered water molecule (Fig. 4A). This water molecule formed bridging H-bonds between the O^γ^-hydroxyl group of T80 and the carboxyl terminus and S8 of the peptide as well as to N77 (HLA). Furthermore, the water molecule forms van der Waals contacts with L166 (KIR), coincident with a 0.5 Å shift in the side chain of L166. The replacement of direct contacts at the binding interface with a network of water-mediated contacts will subtly affect the dynamics of the interaction and likely accounts for the reduction in affinity for Bw4 80T allotypes observed with 3DL1*001.

Next, we examined the role of peptide in the context of HLA Bw4 80T using a panel of three HLA-B*27:05 tetramers (Table II). In addition to the HIV-derived KK10 peptide, we also examined the binding to the HBV-derived KF9 (KRWGYSLNF) epitope and its variant KF9 N8H (KRWGYSLHF). In contrast to HLA-B*57:01 tetramer binding, mutation of 3DL1 residues M165 or L166 generally decreased the HLA-B*27:05 interaction; the exception was HLA-B*27:05-KK10, which displayed increased binding in the presence of L166A (Fig. 4B). The role of A167 also appeared to be peptide-dependent because the A167G mutant showed decreased binding to both the KK10 and KF9 tetramers, but not to the KF9-H8 variant. These findings uncover a role for the 165–167 loop in controlling HLA–Bw4 80T interactions that is dependent on the sequence of the presented peptide and distinct from that seen with HLA-Bw4 80I. Thus, variation in both the peptide sequence and the Bw4 motif alters the contribution of individual KIR contact residues to the overall stability of the KIR–HLA complex.

**FIGURE 5.**
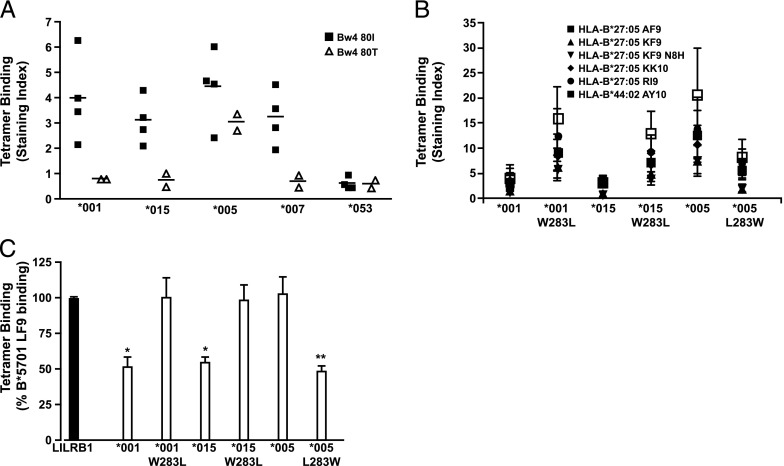
Position 283 controls 3DL1 allotypic discrimination between Bw4 80I and 80T. (**A** and **B**) HEK293 cells were transfected with FLAG-tagged 3DL1 allotypes or mutants thereof with reverted amino acid substitutions at position 283, then stained with individual Bw4 80T tetramers (0.2 μg each) as indicated. (**C**) 3DL1 allotypes or position 283 mutants were stained with HLA-B*57:01.I80T-LF9 tetramer (0.2 μg). Data are shown as percentage binding relative to the wild-type HLA-B*57:01-LF9 tetramer. Binding to LILRB1-expressing cells is shown as a control. Data are averaged from at least *n* = 3 independent experiments. *p < 0.05, **p < 0.01. Statistical significance was assessed by ANOVA with a Tukey multiple comparison posttest. Error bars represent SEM.

The interaction of certain 3DL1 allotypes showed a strong preference for Bw4 80I over Bw4 80T interactions, whereas others seemed to interact equally well with both classes of Bw4 molecules (Fig. 5A). Despite some suggestions from genetic association studies, we found that this discriminatory capacity did not segregate well with low and high KIR allotypes. For example, *007 showed a pattern more similar to the high allotypes *001 and *015 than to its fellow low allotype *005. The binding of *053 (a low allotype) to both Bw4 80I and 80T was poor and nonpreferential (Fig. 5A). Guided by this and by our peptide interaction results (Figs. 2, 3), we examined the role of residue 283 in the discrimination between HLA Bw4 subgroups. Position 283 was completely responsible for the preferential binding of Bw4 80I over Bw4 80T in W283 allotypes. The W283L mutation in both *001 and *015 substantially increased their interactions with all members of a panel of Bw4 80T tetramers (Fig. 5B). Conversely, *005 showed much reduced recognition of Bw4 80T in the presence of the L283W mutation. In contrast, no consistent effect of substitution at position 283 was observed on Bw4 80I tetramer binding (data not shown). To confirm the role of position 80, we used HLA-B*57:01-LF9 tetramers containing the I80T mutation. As expected, *001 (and *015) showed decreased binding to HLA-B*57:01 I80T tetramers, whereas *005 showed no ability to discriminate between Bw4 80I and 80T. This effect could be reversed for all three allotypes by switching residues at KIR position 283 (Fig. 5C). Thus, the bifurcation of 3DL1 variants based on this D2 residue represents the emergence in humans of an allotype lineage (*005-like) that exhibits both broader peptide recognition and broader HLA Bw4 recognition.

## Discussion

In this study, we describe a dichotomy within the 3DL1 family of KIRs based on position 283 in the D2 domain that confers distinct patterns of HLA reactivity and peptide permissiveness. Two groups of allotypes, corresponding to the *015-like and *005-like genetic lineages, are delineated by these data. The *005*-*like lineage, incorporating leucine at position 283, shows robust recognition of both Bw4 80T and 80I HLA allotypes and broader peptide tolerance than the *015-like lineage, which incorporates tryptophan at the corresponding site. Thus, we define a molecular signature that underlies a functional bifurcation of 3DL1 allotypes, thereby providing the basis for a simple typing assay that would be readily applicable to clinical studies.

For both the LF9-E8 and TW10-D9 peptides, our findings suggest that charged residues, in particular negatively charged residues, disturb the interaction with 3DL1. These data are in line with the findings of Stewart-Jones et al. (20), who found that glutamic acid at P8 was not tolerated in the context of HLA-B*27:05. Our data suggest that this reflects the role of KIR residue E282 in the peptide interaction. This negatively charged residue clearly restricts tolerance of negatively charged C-terminal peptide residues. However, perturbation of E282 can result in unique peptide specificity profiles, suggesting that its role in peptide discrimination is not simply due to the potential for electrostatic clashes. During immune-mediated evolution of HIV epitopes within a particular host, the acquisition of negatively charged C-terminal residues may interfere with 3DL1*001 recognition and result in NK cell activation, to the detriment of the virus. Our data suggest that L283 allotypes, including *005, are less sensitive to these peptide substitutions, and these donors are more likely to maintain NK cell inhibition. Accordingly, we would predict commensurate increases in the frequency of such HIV-derived epitope mutations.

The dimorphic residue at position 283 has a dramatic influence on both peptide and HLA interactions. This could reflect either a direct role in the peptide-laden HLA interaction or transmitted effects impinging on neighboring or distant residues. The increased recognition of the TW10-D9 peptide observed with L283 allotypes is more parsimoniously explained by an effect on the neighboring E282 residue, which is the primary block to TW10-D9 recognition. This same change (W283L) also results in equivalent recognition of Bw4 80I and 80T molecules. In this case, it is possible that the positioning or availability of E282 results in increased affinity for Bw4 80T molecules. However, structural analysis reveals that the side chains of these two residues (282 and 283) are not in contact. Thus, it is perhaps more likely that position 283, located at the interface between the D1 and D2 domains, influences the relative positioning of these two domains. The switch between the large, bulky, hydrophobic tryptophan and the smaller leucine residue would be expected to influence the hinge angle between these domains and thereby influence ligand interactions. Indeed, the differential interaction of 2DL2 and 2DL3 with HLA-C2 allotypes is thought to result from an influence of polymorphic residues on the interdomain hinge angle (34, 35). A role for position 283 in 3DL1 protein conformation has also been suggested by Parham and colleagues (36) on the grounds that this amino acid results in differential Ab affinity.

The division of 3DL1 allotypes based on a dichotomy at position 283 defines two separate lineages with distinct peptide and Bw4 80T epitope recognition properties. In all other KIR molecules, including 3DS1, 3DL2, 2D, and nonhuman primate KIRs, the position equivalent to 283 is represented by tryptophan. Genetic analysis of the 3DL1 lineages suggests that both L283 and W283 are ancient and actively maintained in human populations, albeit with some exceptions (4). The emergence and maintenance of L283 implies a functional advantage at the population level to the presence of both 3DL1 specificities. In light of the known coevolution of HLA and KIR (37), it is interesting to speculate that the *005-like lineage might have arisen in populations with a high prevalence of Bw4 80T. In these populations, W283 3DL1 allotypes would be expected to lead to poor recognition, and the emergence of L283 would have generated a 3DL1 specificity capable of robust interactions with the dominant HLA allotypes.

In a functional analysis of the interaction of KIR3DL1 allotypes with a broad array of HLA-Bw4–expressing target cells, Foley et al. (38) did not detect any discrimination between 80I and 80T allotypes using 3DL1*001, *005, *015, and *053 allotypes. Based on our binding data, we would have predicted that although *005 and *053 (L283) would not discriminate, both *001 and *015 would show a preference for 80I Bw4 allotypes. Indeed, other functional assays have revealed a preference for 80I by some KIR3DL1 allotypes, including *001 (8, 13), and epidemiological evidence from HIV infection supports a functional role for the Bw4 80I/80T dichotomy in KIR interactions that ultimately influence disease outcome. Taken together, these findings suggest that the differences in binding affinities that we describe in this study do have functional consequences. Although these differences may be masked in some experimental set-ups (due, for example, to high levels of ligand expression, expression of additions receptors, and other factors), there is an abundance of data that suggest the groupings we define in the present study are functionally relevant and should be considered in the clinical assessment of KIR–HLA interactions.

The most common functional allotype within the L283 grouping is *005. This allotype is found at a frequency of ∼10–40%, although it is quite rare in some populations (39). Other L283 allotypes include *004 (which is not expressed at the cell surface), *019, *040, *041, *042, and *053; with the exception of *004, however, these are not found at high frequencies in human populations. Thus, the dominant L283 allotype (*005) is also a low 3DL1 variant. The functional consequences of low expression are not clear. In an in vitro assay, NK cells expressing the high *002 or the low *005 receptors were equally inhibited by a target cell line expressing the Bw4 80I allotype B*58:01 (40). It is possible that during their maturation process, NK cells are tuned to the level of inhibitory signal received. In this scenario, NK cells expressing low levels of 3DL1 are maximally inhibited by a lower signal threshold than those expressing higher levels of 3DL1. This is similar to the education model, in which those NK cells that do not receive any inhibitory signals are hypofunctional, an effect that has been shown to exist across a spectrum of reactivities according to the rheostat model (41, 42).

Genetic association studies that have classified 3DL1 receptors into high and low on the basis of expression, and have found differential interactions with Bw4 80T allotypes, may therefore simply reflect a molecular division that hinges on the residue present at position 283. As such, low allotypes would show better epistatic interactions with Bw4 80T allotypes solely due to the dominant role of *005 (a L283 allotype) in Bw4 80T recognition. The next most common low allotype is *007, which incorporates tryptophan at position 283. By our predictions, it should segregate with high allotypes due to the common residue at position 283.

In summary, we have described a lineage-defining dimorphism in 3DL1 allotypes that controls both HLA reactivity and peptide specificity. The categorization of KIR-HLA combinations into functional groups is of significant clinical interest in multiple settings. At present, the classification of KIR allotypes into high or low expression requires complete 3DL1 subtyping and is based purely on phenotypic evaluation because there are no common molecular features that segregate with all low allotypes. Some of the amino acids responsible for low cell surface expression have been identified for some allotypes (e.g., *005, *028) (11, 43), but these are not shared with other low allotypes (e.g., *007). In contrast, the classification of allotypes based on the amino acid present at position 283 is straightforward and definitive, and we are currently developing a simplistic typing system that would provide quantitative information on the possession of 3DS1, 3DL1*004, 3DL1 L283, and 3DL1 W283 allotypes. This assay will enable direct measurement of the causative variant in the functional bifurcation of 3DL1 allotypes and should prove amenable to rapid and cost-effective clinical implementation.
